# Systematic evaluation of DNA methylation age estimation with common preprocessing methods and the Infinium MethylationEPIC BeadChip array

**DOI:** 10.1186/s13148-018-0556-2

**Published:** 2018-10-16

**Authors:** Lisa M McEwen, Meaghan J Jones, David Tse Shen Lin, Rachel D Edgar, Lucas T Husquin, Julia L MacIsaac, Katia E Ramadori, Alexander M Morin, Christopher F Rider, Chris Carlsten, Lluís Quintana-Murci, Steve Horvath, Michael S Kobor

**Affiliations:** 1 BC Children’s Hospital Research Institute, Department of Medical Genetics, University of British Columbia, 950 West 28th Avenue, TRB A5-151, Vancouver, BC V5Z 4H4 Canada; 2 Department of Medicine, Division of Respiratory Medicine, University of British Columbia, Vancouver, BC Canada; 3 Unit of Human Evolutionary Genetics, Institut Pasteur, 75015 Paris, France; 4 Centre National de la Recherche Scientifique (CNRS) UMR2000, 75015 Paris, France; 5 Center of Bioinformatics, Biostatistics and Integrative Biology, Institut Pasteur, 75015 Paris, France; 6 Department of Human Genetics, David Geffen School of Medicine, University of California, Los Angeles, CA USA

**Keywords:** Epigenetic age, DNA methylation age, Epigenetic clock, EPIC, DNA methylation, 450K, Human, Microarray, Preprocessing

## Abstract

**Background:**

The capacity of technologies measuring DNA methylation (DNAm) is rapidly evolving, as are the options for applicable bioinformatics methods. The most commonly used DNAm microarray, the Illumina Infinium HumanMethylation450 (450K array), has recently been replaced by the Illumina Infinium HumanMethylationEPIC (EPIC array), nearly doubling the number of targeted CpG sites. Given that a subset of 450K CpG sites is absent on the EPIC array and that several tools for both data normalization and analyses were developed on the 450K array, it is important to assess their utility when applied to EPIC array data. One of the most commonly used 450K tools is the pan-tissue epigenetic clock, a multivariate predictor of biological age based on DNAm at 353 CpG sites. Of these CpGs, 19 are missing from the EPIC array, thus raising the question of whether EPIC data can be used to accurately estimate DNAm age. We also investigated a 71-CpG epigenetic age predictor, referred to as the Hannum method, which lacks 6 probes on the EPIC array. To evaluate these epigenetic clocks in EPIC data properly, a prior assessment of the effects of data preprocessing methods on DNAm age is also required.

**Methods:**

DNAm was quantified, on both the 450K and EPIC platforms, from human primary monocytes derived from 172 individuals. We calculated DNAm age from raw, and three different preprocessed data forms to assess the effects of different processing methods on the DNAm age estimate. Using an additional cohort, we also investigated DNAm age of peripheral blood mononuclear cells, bronchoalveolar lavage, and bronchial brushing samples using the EPIC array.

**Results:**

Using monocyte-derived data from subjects on both the 450K and EPIC, we found that DNAm age was highly correlated across both raw and preprocessing methods (*r* > 0.91). Thus, the correlation between chronological age and the DNAm age estimate is largely unaffected by platform differences and normalization methods. However, we found that the choice of normalization method and measurement platform can lead to a systematic offset in the age estimate which in turn leads to an increase in the median error. Comparing the 450K and EPIC DNAm age estimates, we observed that the median absolute difference was 1.44–3.10 years across preprocessing methods.

**Conclusions:**

Here, we have provided evidence that the epigenetic clock is resistant to the lack of 19 CpG sites missing from the EPIC array as well as highlighted the importance of considering the technical variance of the epigenetic when interpreting group differences below the reported error. Furthermore, our study highlights the utility of epigenetic age acceleration measure, the residuals from a linear regression of DNAm age on chronological age, as the resulting values are robust with respect to normalization methods and measurement platforms.

**Electronic supplementary material:**

The online version of this article (10.1186/s13148-018-0556-2) contains supplementary material, which is available to authorized users.

## Background

Epigenetics is a rapidly evolving field in the contexts of new biological discoveries as well as the available technologies used to drive such findings. The most commonly studied epigenetic mark in humans is DNA methylation (DNAm), defined as the covalent addition of a methyl group to DNA, most frequently occurring at cytosine-guanine dinucleotides (CpGs) [20]. DNAm profiles change naturally during the development of an organism, resulting in tissue identity being the strongest predictor of DNAm variation. As such, DNAm variability between tissues, for example, the blood and brain, within an individual can be larger than the variability observed across individuals from the same tissue [7, 27]. Inter-individual variability in DNAm has been linked to a number of different sources, including but not limited to the underlying DNA sequence, environmental exposures, and health outcomes. One of the most active areas of research related to DNAm inter-individual variability in human cohorts focuses on the relationship between DNAm and aging, as there has been substantial evidence that DNAm changes with age, both linearly and non-linearly, across the entire life course [12].

Rapidly evolving new technologies and resources have fueled exponential growth in human DNAm research over the past decade, enhancing our ability to address questions such as the effects of aging. Although many methodologies can be used to measure DNAm, Illumina microarrays are the most common method for population-based epigenetic studies, as they provide an economical and accessible high-throughput platform. Over a little more than a decade, the capacity of Illumina DNAm microarray platform has increased from 1506 CpGs to more than 860,000 CpGs. The increased numbers of CpGs reflect both better coverage across genes and expanded interrogation of genomic regions. For example, the Illumina 27 K (27 K) array targeted > 27,000 CpG sites and interrogated at least one CpG per gene, but was biased towards CpG islands [2]. Its successor, the Illumina Infinium Methylation450 (450K) array assessed > 485,000 CpGs and covered 99% of RefSeq genes. The Illumina Infinium MethylationEPIC (EPIC) array is the newest tool and allows the quantification of over 860,000 CpG sites, with the additional content providing higher coverage of specific genomic regions, such as enhancers and non-coding regions. The EPIC array generally uses the same DNAm measurement protocol as the 450K array and includes over 94% of the 450K content [24]. However, the increased genomic resolution and complexity of the EPIC array in conjunction with missing 6% of the 450K CpGs necessitates an evaluation of the applicability of established bioinformatic tools established for the 27 K or 450K arrays.

To accommodate advancements in DNAm array technology and the increasing volume of data, many pipelines for data preprocessing, normalization, and analyses have been developed to streamline data handling [1, 5, 25, 28, 30]. Here, we refer to “preprocessing methods” as algorithms commonly performed on DNAm data prior to probe-type normalization, including methods to reduce background fluorescence or adjust for dye bias, which if unaddressed can reduce the dynamic range of beta values [31]. Probe-type normalization is a necessary adjustment for Illumina microarray DNAm data, as there are two different probe designs that possess differential beta distributions [2]. Tools such as the R function ‘preprocessNoob’ in the minfi package subtract background based on the out-of-band intensities (for example, Infinium I probes fluorescing in the color channel opposite their designed base extension). Color or dye bias adjustment is applied to account for the two color channels that type II probes employ, one for methylated and one for unmethylated CpGs, since residual dye can introduce unwanted variation. Tools to account for the color bias include in the Bioconductor package ‘methylumi,’ which is based on smooth quantile normalization, or the Illumina GenomeStudio software which implements a shift-and-scaling normalization [6]. Although these methods have been reviewed in comparison to one another [33, 35, 36], a mixed variety of pipelines are used across the literature and the influence of method selection on detecting true positives or generating accurate predictions should be investigated both within and across array technologies.

One tool that could be compromised by different preprocessing methods or the lack of certain 450K CpG sites on the EPIC array is the pan-tissue epigenetic clock, a popular predictive model that estimates an individual’s biological age, irrespective of tissue type, using DNAm at 353 CpGs [12]. Established on DNAm profiles (obtained from 27 K and 450K data) from 51 different tissues from over 8000 individuals, the epigenetic clock calculates DNAm age, which has been shown to correlate well with chronological age (*r* > 0.80) across the life course [14]. This epigenetic clock is hypothesized to be an accurate molecular biomarker of biological aging and deviations between chronological and DNAm age, commonly referred to as epigenetic age acceleration (which can be positive or negative), have been correlated with a host of age-related conditions, such as Parkinson’s disease, time until death, frailty, and cognitive and physical decline [4, 15, 22, 23].

There are several other DNAm-based age predictors that have been reported [3, 9, 34], but another commonly used age predictor, specific to blood samples and referred to as the Hannum clock, is based on methylation at 71 CpG sites has also been observed to predict age with impressive accuracy. However, both the Horvath and Hannum models are lacking CpG sites on the on the EPIC array (19 of the 353 pan-tissue clock-CpGs and 6/71 of the Hannum clock CpGs are missing), and since the 450K platform is no longer available, it is crucial to assess the performance of these tools despite the missing probes, if use is to be continued.

Here, we investigated (1) the consistency between DNAm age measured from 450K and EPIC array data from the same individuals to evaluate the utility of EPIC array data given that it is missing clock CpGs used in the Horvath and Hannum age predictors, and (2) whether DNAm age estimates differ with different preprocessing methods. We found that EPIC data can be used to predict DNAm age accurately using both assessed epigenetic clocks. Additionally, we observed differences in DNAm age across preprocessing methods, although the differences across the values were below the reported median absolute error of the epigenetic clock. Lastly, we have replicated accurate measurement of DNAm age, using the pan-tissue predictor, across tissues using an EPIC dataset with three different tissues from 13 individuals. Our findings support the epigenetic clock as a robust tool that may be applied with EPIC array data in the future.

## Methods

### Cohort characteristics

We used two different cohorts in order to assess the pan-tissue epigenetic clock on the EPIC array. The first consisted of primary monocytes collected from 172 healthy males, aged 19–50 years old, of self-reported African- and European-descent from the EVOIMMUNOPOP project [19]. Genomic DNA was isolated from the monocyte fraction using a phenol/chloroform protocol followed by ethanol precipitation, and then subjected to bisulfite conversion with the EZ DNA Methylation Kit (ZymoResearch, Irvine, CA, USA). We quantified DNAm on all samples using two separate Illumina microarray platforms: 450K and EPIC arrays (Illumina, San Diego, CA, USA), following the manufacturer’s instructions. To ensure sample labeling across technologies, we assessed the correlation between the overlapping quality control single-nucleotide polymorphic (SNP) probes present on both microarrays (59 SNPs); observing all sample pairs correlated with a Pearson’s coefficient of *r* ≥ 0.99 (Additional file 1: Figure S1). Four technical replicates were included during the 450K processing and 12 technical replicates were included during the EPIC sample processing, with two common technical replicates across technologies.

A secondary cohort was used to investigate EPIC DNA methylation data derived from tissues other than the blood. This cohort consisted of 13 individuals aged 23–46 years old from the control subset of Diesel Exhaust Study III (DE3) and was comprised of DNAm from peripheral blood mononuclear cells (PBMCs), bronchoalveolar lavage (BAL), and bronchial brushings (brush). All samples were collected from individuals after control (filtered air/saline) exposures. Primary cohort characteristics are provided in Additional file 1: Table S1. Note, approximately half of the individuals had prior physician-diagnosed asthma. Genomic DNA was isolated using the DNeasy Blood and Tissue Kit (Qiagen, Hilden Germany) and subsequently bisulfite converted using the EZ DNA Methylation Kit (ZymoResearch, Irvine, CA, USA). Bisulfite-treated samples were processed using the EPIC array as above (Illumina, San Diego, CA, USA).

### DNA methylation quantification

All microarrays were scanned with an Illumina HiScan system. For the EPIC array data, we used the most current manifest file, “Infinium MethylationEPIC v1.0 B4 Manifest File,” released by Illumina on May 26, 2017 and consisting of 865,918 probes, whereas for the 450K we used the “HumanMethylation450 v1.2 Manifest File” with 485,577 probes. Both manifest files are available at https://support.illumina.com/downloads.html. In addition to unprocessed (raw) data, we used data preprocessed in three different ways (1) color corrected/background subtracted in Genome Studio (GS), (2) quantile-normalized using “preprocessQuantile” [29], (3) normal-expontential out-of-band (noob)-normalized with “preprocessNoob” [30]. Raw data and data that were to be quantile or noob normalized were uploaded directly into R from IDAT files using the ‘minfi’ package function “read.metharray” [8]. For the color correction/background subtracted preprocessing, data were background subtracted/color corrected with GenomeStudio, and then uploaded into R with the package ‘methylumi,’ function ‘lumiMethyR’ [5].

### DNA methylation age

We calculated DNAm age for each sample by using a modified version of the publicly available R code at https://dnamage.genetics.ucla.edu, with the normalization feature set to “TRUE” [12]. We focused our inquiry on data preprocessing only, and not probe-type normalization methods, as the epigenetic clock code applies an imputation of missing values and performs a calibrated version of a beta-mixture quantile normalization [12, 28]. The 71-CpG Hannum method age estimates were generated using methods described previously [10].

## Results

### The epigenetic clock accurately predicted DNA methylation age from EPIC methylation data

From the 450K array, 33,059 of 485,557 (6.8%) of probes are not represented on the EPIC array, including 19/353 epigenetic clock-CpGs (5.4%). The lack of 19 epigenetic clock CpGs on the EPIC array could reduce the accuracy of the epigenetic clock when using EPIC array data. Therefore, we investigated the consistency between DNAm age as calculated from the 450K (original 353-CpG model) and the EPIC (reduced 334-CpG model) arrays.

**Fig. 1:**
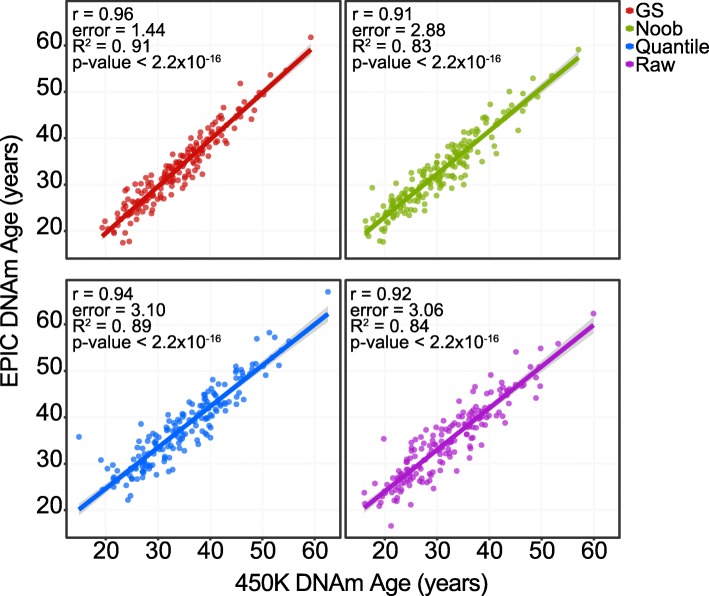
DNA methylation age comparison between 450K or EPIC Monocyte data across preprocessing methods. Identical samples were assayed on both the 450K and EPIC arrays, and then each preprocessed in one of four ways prior to calculating DNA methylation (DNAm) age: raw unprocessed, GenomeStudio color correction/background subtraction (GS), normal exponential out-of-band (noob) normalization, or quantile normalization. Solid colored line represents corresponding group regression line. For each regression, the Pearson’s correlation coefficient, error (median absolute error between EPIC DNAm age and 450K DNAm age), *R*^2^ value, and *p* value corresponding to the correlation coefficient are shown

Focusing on a recently published data set of DNAm in purified monocytes from the EVOIMMUNOPOP project [19], we applied the epigenetic clock to data from samples run on both platforms and found a high correlation between the 450K and EPIC array DNAm age values regardless of preprocessing method (*r* = 0.91–0.96, error = 1.44–3.10 years, *R*^2^ = 0.83–0.91, Fig. 1), observing consistent patterns between chronological age and DNAm age as measured from both the EPIC (*r* = 0.84–0.86) and 450K (*r* = 0.86–0.87) arrays (Additional file 1: Figure S2). Additionally, we performed probe-wise correlations of log transformed beta values at the 334 common clock CpG sites across the two platforms and found a range of Pearson’s correlation coefficients *r* = − 0.18–0.98 (Additional file 1: Figure S3A); specifically, out of the 334 probes an average (across preprocessing data sets) of 146 (44%) had ≤ 0.20, 118 (35%) had *r* > 0.50, and 44 (13%) had *r* > 0.80. Previous reports showed low correlation associated with low variation across the EPIC and 450K arrays, and so we tested to determine whether the clock probes with low correlation were also invariable. We observed that lower beta value ranges were strongly associated with lower correlation values between the EPIC and 450K (*r* = 0.75–78, depending on preprocessing method, Additional file 1: Figure S3B). As a second approach to assess the direct consequence of the absent clock sites, we removed the 19 missing EPIC clock-CpGs in the 450K data to simulate the 334-CpG model. We then calculated DNAm age using both the 353-CpG model and the 334-CpG model from the 450k data, finding a strong correlation of *r* = 0.998, indicating that the missing 19 CpGs did not adversely affect DNAm age prediction in monocytes (Additional file 1: Figure S4). Furthermore, we calculated another DNAm age measure based on the Hannum method using 71 CpG sites of which only 6 (8.5%) are missing on the EPIC array [10], and again found strong correlations between Hannum DNAm age as calculated from EPIC and 450K array data (*r* = 0.92–0.95, Additional file 1: Figure S5).

**Fig. 2:**
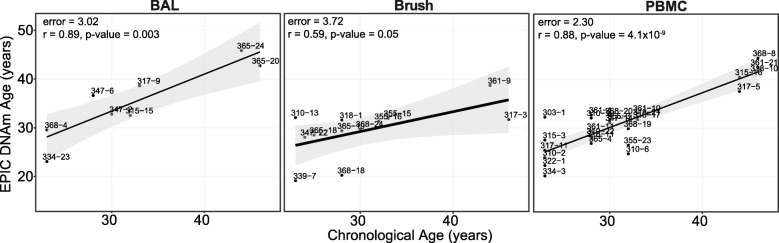
EPIC DNA methylation age estimated in control samples from the Diesel Exhaust III Study across three tissues. DNA methylation (DNAm) age was estimated using the EPIC 334-CpG model from GenomeStudio background-subtracted and color-channel-adjusted EPIC data. Linear regression line shown with 95% confidence intervals is shown in gray. Error is the median absolute difference between EPIC DNAm age and chronological age. Pearson’s correlation coefficients (*r*) and corresponding *p* value are shown for each tissue. BAL = bronchoalveolar lavage, PBMC = peripheral blood mononuclear cells, brush = bronchial brushing

Lastly, to confirm that the epigenetic clock could produce an accurate estimate of age from EPIC data across different tissues, we used an independent cohort of three tissues (PBMCs, BALs, brushes) collected from 13 healthy adults. Importantly, data from lung tissues has been reported previously to accurately estimate DNAm age in the context of DNAm age using the 450K array [12]. Calculating DNAm age with GS-preprocessed data, we observed a strong correlation with chronological age using EPIC data for the PBMC and BAL samples (*r* = 0.88, *r* = 0.89, respectively), but a lesser degree of correlation for the brush samples (*r* = 0.59, Fig. 2). We note that the brush beta-value distribution appeared to have higher inter-individual variability than the other tissues, which may explain the lower correlation in brush samples (Additional file 1: Figure S6).

### Data preprocessing methods affected the calculated DNA methylation age, but within error margins of the epigenetic clock

**Fig. 3:**
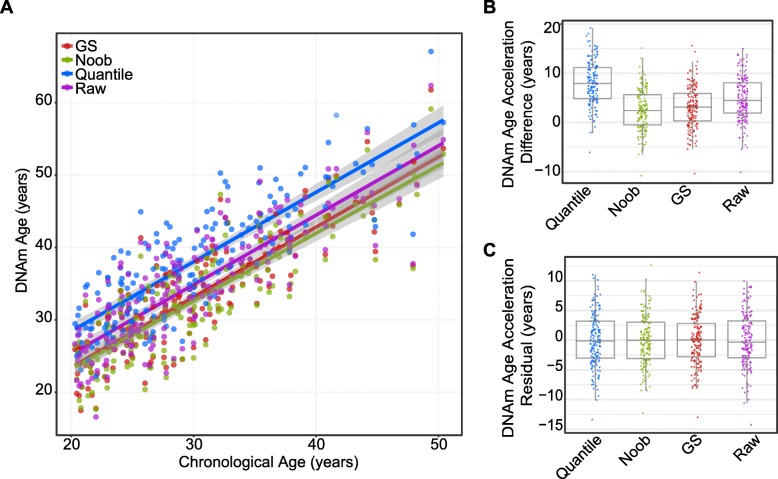
DNA methylation age acceleration variation across preprocessing methods. **a** Scatter plot of EPIC DNA methylation (DNAm) age calculated from raw and data from three different preprocessing methods: quantile, GenomeStudio (GS), and normal exponential out-of-band (noob) normalization. Colored regression lines and surrounding shaded gray areas represent 95% confidence interval for each group. **b** Boxplot of estimated DNA methylation (DNAm) age—chronological age (acceleration difference) for each preprocessing method. **c** Boxplot of residuals from a linear regression (DNAm age~chronological age) across methods. The median is illustrated by horizontal line with upper and lower hinges representing the 25th and 75th percentiles, upper and lower whiskers extend no further than the inter-quartile range multiplied by 1.5. Colored data points represent individual samples for each group

Given that there is not an accepted standard practiced method of preprocessing data prior to calculating DNAm age, we assessed the potential effects of different commonly used data preprocessing methods on the DNAm age estimates. We compared DNAm age estimates calculated from raw data as well as after applying three separate standard data preprocessing methods: color-correction and background-normalization with GenomeStudio software (abbreviated GS), quantile-normalization, or noob-normalization. Imputation and a probe-type normalization were performed the same way across preprocessing methods using the R code supplied with the epigenetic clock method [16, 28, 32]. Using monocyte-derived data from 172 subjects on both the 450K and EPIC, we found that DNAm age was highly correlated across both raw data and data after three different preprocessing methods (*r* > 0.91) (Additional file 1: Figure S2). However, shifts in mean DNAm age were observed, indicating that although mean DNAm differences did exist, the trends with age were consistent across preprocessing methods (Fig. 3a). This was further supported by significant Kendall rank coefficients in DNAm age across each preprocessing method (τ = 0.86–0.94, *p* value < 2.2 × 10^− 6^, Additional file 1: Figure S7).

To further investigate the sample-to-sample trend in DNAm age across methods, we explored two common measures associated with the epigenetic clock, both considered measures of epigenetic age acceleration; the difference between DNAm age and chronological age (age acceleration difference) and the residuals from a linear model of DNAm age regressed onto chronological age (age acceleration residual). Since the observed mean DNAm age shifts when using different preprocessing methods (Fig. 3a), age acceleration difference is more likely to be affected by which preprocessing method was chosen. In contrast, age acceleration residual is less affected by mean differences as it is expressed relative to the measured population. As expected, we observed significant discrepancies in the mean age acceleration difference measure for nearly all comparisons (*p* value < 0.0002 for all age acceleration difference comparisons except for noob versus GS *p* value = 0.23, median absolute difference ranging from 0.68–5.55 years (Fig. 3b, Additional file 1: Table S2). Minimal variation was observed for the age acceleration residual mean across preprocessing methods (*p* value > 0.99, median absolute difference ranging from 0.52–1.23 years, Fig. 3c, Additional file 1: Table S2). This supports the previous suggestion of using age-acceleration residuals [12] to correct for processing specific shifts in DNAm estimates in order to accurately compare DNAm age between people.

**Fig. 4:**
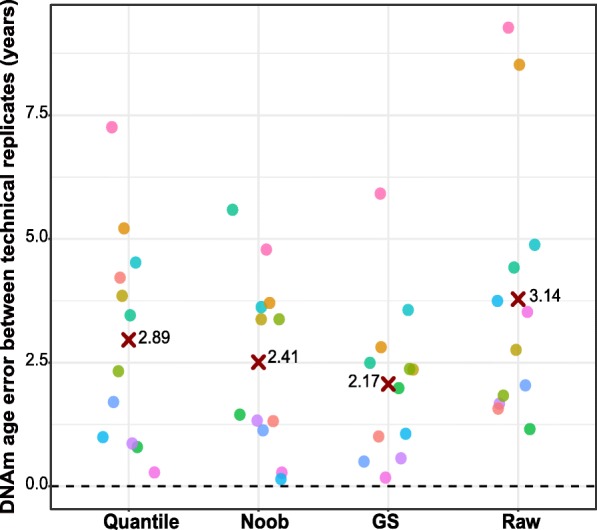
Absolute difference between technical replicate pairs for each processing method. The *y* axis represents the absolute difference between each technical replicate pair’s DNA methylation (DNAm) age. Each processing method is represented on the *x* axis. The median difference is indicated by the red cross for each group. The color of data points represents one technical replicate pair for ease of interpretation across methods. Error refers to the median absolute difference between DNAm age and chronological age

We assessed how different preprocessing methods influenced the DNAm age estimate by examining the concordance of DNAm age measured from EPIC array technical replicates. A technical replicate pair represented an identical DNA sample quantified twice for quality control purposes; specifically, the sample was divided into two separate tubes after bisulfite conversion and DNAm was quantified separately. Technical replicate sample identity was confirmed by examining the 59 SNP probes present on the EPIC array (Additional file 1: Figure S8). We focused on the 24 technical replicates (12 pairs) from the EPIC array, calculating DNAm age for each technical replicate from data subjected to each separate preprocessing method: raw, GS color corrected and background subtracted, quantile normalized, or noob normalized. We calculated the median absolute difference between each technical replicate pair’s DNAm age estimates in each dataset. We found that the GS color correction and background subtraction had the least deviation across replicates (error_GS_ = 2.17 years), followed by noob normalization (error_Noob_ = 2.41 years), quantile normalization (error_Quantile_ = 2.89 years), and then raw data having the largest deviation (error_Raw_ = 3.14 years, Fig. 4). Notably, these values are all below the median absolute error of the epigenetic clock (3.6 years) [12].

## Discussion

This study had two primary aims (1) to investigate whether using EPIC methylation data to calculate DNAm age is an appropriate approach, given the 19 and 6 missing probes used in the pan tissue 353 CpG model (Horvath) and 71 CpG model (Hannum), respectively, and (2) to evaluate the effect of various data preprocessing methods prior to calculating DNAm age, as a standard pipeline for processing data prior to calculating DNAm age does not exist. By analyzing monocyte DNAm from 172 individuals quantified on both the 450K and EPIC arrays, we demonstrated that the lack of the clock-CpGs on the EPIC array did not compromise the utility of the epigenetic age predictors. We also evaluated the performance of the EPIC pan-tissue epigenetic clock (334-CpG model) on another EPIC dataset, consisting of three tissues from 13 individuals, finding comparable correlations with age to those reported with the 450K array (353-CpG model). Furthermore, we found small differences in the DNAm age estimate between data preprocessing methods, implying that although the methods assessed here differed in mean values, the trends in respect to chronological age were consistent across methods.

Finding that preprocessing method influenced mean values of DNAm age is important for the interpretation of future analyses, as we demonstrated that variation in DNAm age can be introduced by how the data are preprocessed. Our work here provides supporting evidence for the DNAm age acceleration residual measure, since this value is reflective of inter-individual variability within a measured dataset, and is, therefore, more comparable across studies. In contrast, the DNAm age difference, the crude difference between estimated DNAm age and chronological age, can be reflective of global DNAm shifts due to preprocessing methods.

Whichever measure of DNAm age is used (acceleration difference, residual, or age itself), there is an additional consideration that small effect sizes should be interpreted with caution. To highlight this point, we calculated DNAm age for technical replicates from raw data and three different preprocessing methods. We found that while there was some variability in DNAm age across technical replicates, regardless of preprocessing methods, the observed median absolute error in DNAm age for each method (2.7–3.14 years) was lower than the reported error of the epigenetic clock (3.6 years) [17]. GS-preprocessed data produced the tightest replicates, followed by noob and then quantile normalization, and the consistency between replicates was lowest when DNAm age was calculated from raw data. These findings may suggest using preprocessed data rather than raw data, but overall we emphasize the importance of considering the technical error of the epigenetic clock and caution interpretation of changes of less than 3.6 years.

To examine the appropriateness of using EPIC data to calculate DNAm age for future research, we took advantage of a cohort with DNAm data on the same individuals on both the 450K and EPIC arrays. It is crucial to examine whether the epigenetic clock can continue to be used on EPIC data, as the 450K platform is no longer available. There was high consistency between 450K and EPIC DNAm age estimates, and the lack of 19 CpG sites did not significantly affect the prediction accuracy of the epigenetic clock. Probe-wise correlation coefficients of the 334 common clock CpG sites across the 450K and EPIC were lower than anticipated; however, previous reports have demonstrated that the majority of EPIC probes are not well correlated to those of the 450K and that this is most prevalent at invariable probes [21]. These observations highlight the robustness of the multi-CpG predictors assessed, as despite the low probe-wise correlations the correlation between the estimated ages was highly correlated. This result is consistent with classical test theory in that error for any given probe is random, and largely uncorrelated with the error of other probes, and therefore these random effects would become redundant in a composite index like epigenetic age [26]. The consistency in estimated age lends support to the strength of this predictive model on the EPIC platform and will allow users to continue applying this bioinformatic tool to continue to calculate DNAm age.

To further examine the application of EPIC array data to predict DNAm age, we estimated DNAm age in an independent EPIC array cohort. We observed correlations between EPIC DNAm age and chronological age that were comparable to previous reports, specifically in PBMCs and BAL samples. The strong association in PBMCs is consistent with previous reports of DNAm age in PBMCs as generated from 450K data [13, 18]. We observed less consistency in the brush samples; however, this tissue was not included in the training data of the 353-CpG epigenetic clock and so performance may not be reflective of EPIC array but rather be a property of clock itself. This is reinforced by our experiment removing the 19 clock CpGs not present on the EPIC array from the 450K data, where we observed a nearly perfect correlation with the 353-CpG data, suggesting that the loss of the 19 clock CpG sites did not influence the accuracy of the epigenetic clock.

There are limitations to this study that should be taken into consideration when interpreting the results. The primary datasets we investigated when comparing EPIC versus 450K estimated DNAm age were from monocyte samples, and although we found that the lack of 19 CpGs did not affect the pan-tissue DNAm age estimate in this specific cell type, those 19 CpGs may be important to estimate age in other tissues. Their importance to other tissues remains to be explored. Additionally, the methods applied in the current study should not be generalized across all studies. For example, global normalization methods, such as quantile normalization, are not appropriate in all cases as interesting biological information can be removed in datasets with large variation across samples, such as cancer compared to normal or multiple-tissue projects. Instead, the use of these data transformation methods should be considered on a study-by-study basis [11]. Furthermore, while we are cognizant there are several other available preprocessing options, for the purposes of our exploration and presentation of these data, we only assessed three of the most common methods.

In summary, we have investigated and confirmed that two commonly used methods of DNAm-based age estimation, the 353 CpG Horvath model and the 71-CpG Hannum model, were not compromised when using the latest human DNAm microarray platform, the EPIC array, which is lacking 19/353 CpG and 6/71 CpG targets, respectively. We have also tested whether DNAm age estimates were influenced by the preprocessing stage; for example, whether raw data generated differing results than normalized data. We assessed raw data and three different preprocessed inputs (noob-, quantile-, and GS-normalized) and found age estimates were different, but less than that of the reported error of the model. Related, we finally also provided support for using the age acceleration residual metric rather than the age acceleration difference in studies applying the epigenetic clock. Our work will provide researchers the confidence to investigate DNAm age using the EPIC array, as well as encourage users to critically consider the technical error of the epigenetic clock when interpreting future findings.

## Additional file

Additional file 1: **Table S1.** Cohort characteristics. **Table S2.** Comparisons of age acceleration metrics derived from different data source inputs. **Figure S1.** Correlation heat-map of 59 common polymorphic control probes for 172 common samples run on the EPIC and 450K methylation platforms. **Figure S2.** Correlations between chronological age, 450K DNA methylation age, and EPIC DNA methylation age estimates from each data input. **Figure S3.** Probe-wise correlations of the 334 common clock CpGs across the 450K and EPIC arrays illustrated lower beta range associated lower correlation across platforms for a given CpG. **Figure S4.** Hannum DNA methylation age estimates for 450K (71 CpGs) versus EPIC (65 CpGs) for each preprocessed data type. **Figure S5.** DNA methylation (DNAm) age from a reduced epigenetic age predictor (334 CpGs) compared to the full epigenetic age predictor (353 CpGs) using the same 450K dataset. **Figure S6.** Diesel Exhaust Study III EPIC DNA methylation beta-value distribution across 795,882 sites. **Figure S7.** Heat map of Kendall rank coefficients across preprocessing methods in EPIC data. **Figure S8.** Dendrogram of 59 single nucleotide polymorphic control probes of technical replicates from the EPIC array. (PDF 1168 kb)

## Abbreviations

27 K: Infinium Methylation27 BeadChip
450K: Infinium Methylation450 BeadChip
BAL: Bronchoalveolar lavage
Brush: Bronchial brushing
CpG: Cytosine-phosphate-guanine
DE3: Diesel Exhaust III Study
DNAm: DNA methylation
EPIC: Infinium MethylationEPIC BeadChip
GS: GenomeStudio
Noob: Normal-exponential out-of-band
PBMC: Peripheral blood mononuclear cell
SNP: Single-nucleotide polymorphism
