# Supplementary material for: Systematic evaluation of DNA methylation age estimation with common preprocessing methods and the Infinium MethylationEPIC BeadChip array
Source: Clin Epigenetics. 2018 Oct 16;10:123. doi: 10.1186/s13148-018-0556-2 (PMC6192219; doi:10.1186/s13148-018-0556-2)
Supplement: Supplementary file 1 — Table S1. Cohort characteristics. Table S2. Comparisons of age acceleration metrics derived from different data source inputs. Figure S1. Correlation heat-map of 59 common polymorphic control probes for 172 common samples run on the EPIC and 450K methylation platforms. Figure S2. Correlations between chronological age, 450K DNA methylation age, and EPIC DNA methylation age estimates from each data input. Figure S3. Probe-wise correlations of the 334 common clock CpGs across the 450K and EPIC arrays illustrated lower beta range associated lower correlation across platforms for a given CpG. Figure S4. Hannum DNA methylation age estimates for 450K (71 CpGs) versus EPIC (65 CpGs) for each preprocessed data type. Figure S5. DNA methylation (DNAm) age from a reduced epigenetic age predictor (334 CpGs) compared to the full epigenetic age predictor (353 CpGs) using the same 450K dataset. Figure S6. Diesel Exhaust Study III EPIC DNA methylation beta-value distribution across 795,882 sites. Figure S7. Heat map of Kendall rank coefficients across preprocessing methods in EPIC data. Figure S8. Dendrogram of 59 single nucleotide polymorphic control probes of technical replicates from the EPIC array. (PDF 1168 kb) [file 13148_2018_556_MOESM1_ESM.pdf]

# Supplementary Materials

## Systematic evaluation of DNA methylation age estimation with common preprocessing methods and the Infinium MethylationEPIC BeadChip array

**Authors:** Lisa M. McEwen[1§], Meaghan J. Jones[1], David Tse Shen Lin[1], Rachel D. Edgar[1], Lucas T. Husquin[3], Julia L. MacIsaac[1], Katia E. Ramadori[1], Alexander M. Morin[1], Christopher F. Rider[2], Chris Carlsten[2], Lluis-Quintana Murci[3,4], Steve Horvath[5], Michael S. Kobor[1]

# Tables

**Table S1. Cohort Characteristics**

| | |
|---|---|
| **Monocyte Cohort** | |
| N = | 172 |
| Age (mean[SD]) | 30.0[7.1] |
| Sex (% male) | 100% |
| **Diesel Exhaust Controls** | |
| N = | 13 |
| Age (mean[SD]) | 29.8[7.6] |
| Sex (% male) | 53.9% |

**Table S2. Comparisons of age acceleration metrics derived from different data source inputs.** Age acceleration measures were calculated and compared across each data type (raw, genome studio (GS) colour corrected/ background subtracted, normal-exponential out-of-band (noob) processed, and quantile-normalized data). The age acceleration residual measure was calculated by extracting the residuals of a linear model of DNA methylation age (as predicted from each data type) regressed onto chronological age. The age acceleration difference was calculated by taking the difference between DNA methylation age (as predicted from each data type) and chronological age. Median absolute differences are indicated as first values of each cell and p-values derived from student t-tests are indicated in brackets. All data presented here are from EPIC data. * = $p$-value < 2.2 x $10^{-16}$, NS = $p$-value > 0.99

| | **Raw** | | **Noob** | | **GS** | |
|---|---|---|---|---|---|---|
| | *Residual* | *Difference* | *Residual* | *Difference* | *Residual* | *Difference* |
| **Noob** | 1.23(NS) | 1.99(*) | | | | |
| **GS** | 1.13(NS) | 1.45(*) | 0.52 (NS) | 0.68 (0.23) | | |
| **Quantile** | 1.11(NS) | 3.20(*) | 0.95 (NS) | 5.55 (*) | 0.80(NS) | 5.02(*) |

# Figures

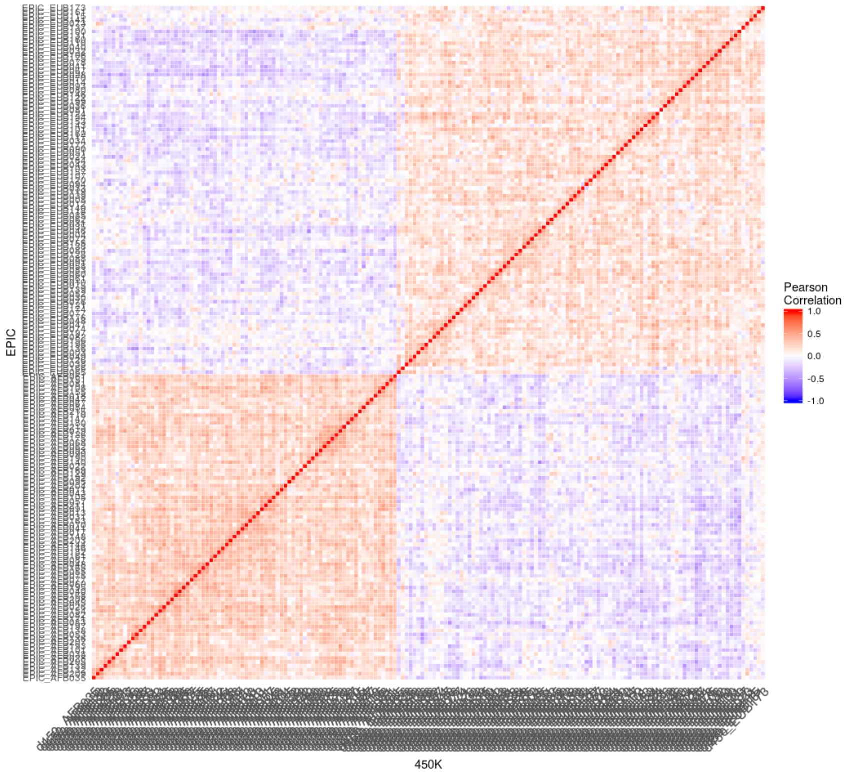


**Figure S1. Correlation heat-map of 59 common polymorphic control probes for 172 common samples run on the EPIC and 450K methylation platforms.**

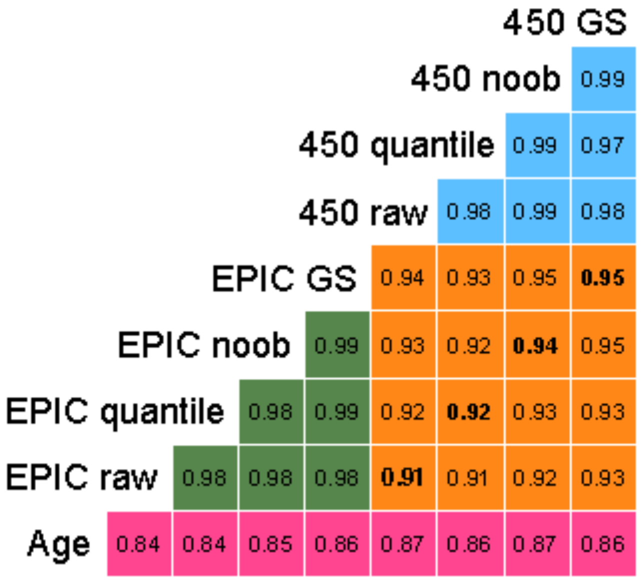


**Figure S2. Correlations between chronological age, 450K DNA methylation age, and EPIC DNA methylation age estimates from each data input.** Green = comparison between EPIC predictions, blue = comparison between 450K predictions, orange = comparison between EPIC and 450K estimates at each processing stage, pink = comparison of each data platform and stage with chronological age. Bold indicates same processing stage correlation between the 450K and EPIC platforms. Pearson's correlation coefficients shown.

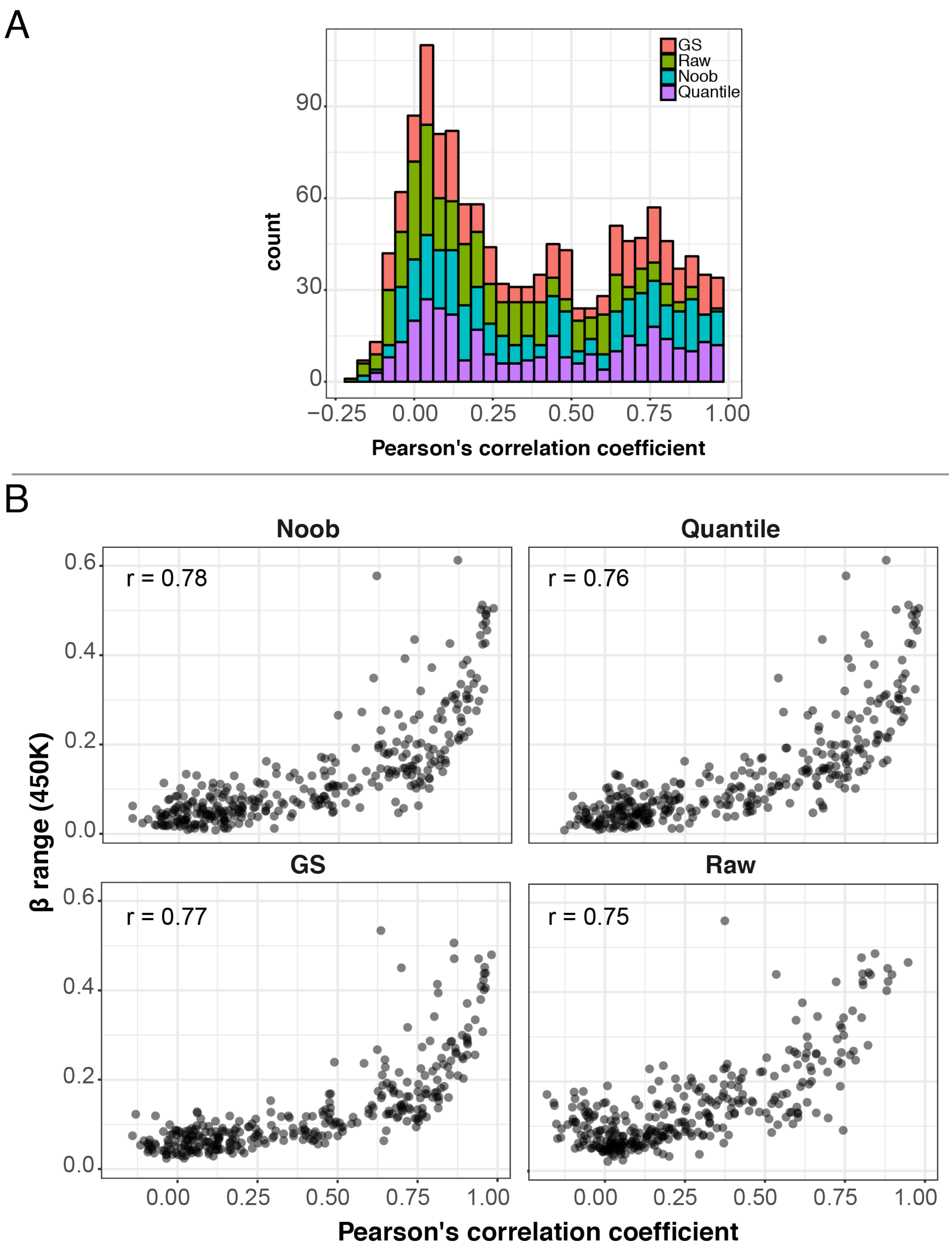


**Figure S3. Probe-wise correlations of the 334 common clock CpGs across the 450K and EPIC arrays illustrated lower beta range associated lower correlation across platforms for a given CpG.** A) Histogram of Pearson correlation coefficients across the 334 CpG sites coloured by different preprocessing method. B) Scatter plots of ß (percent methylation) Range = max(beta) - min(beta) against Pearson’s correlation coefficient for preprocessing method. Note: all probe wise correlations presented were performed on log transformed beta values.

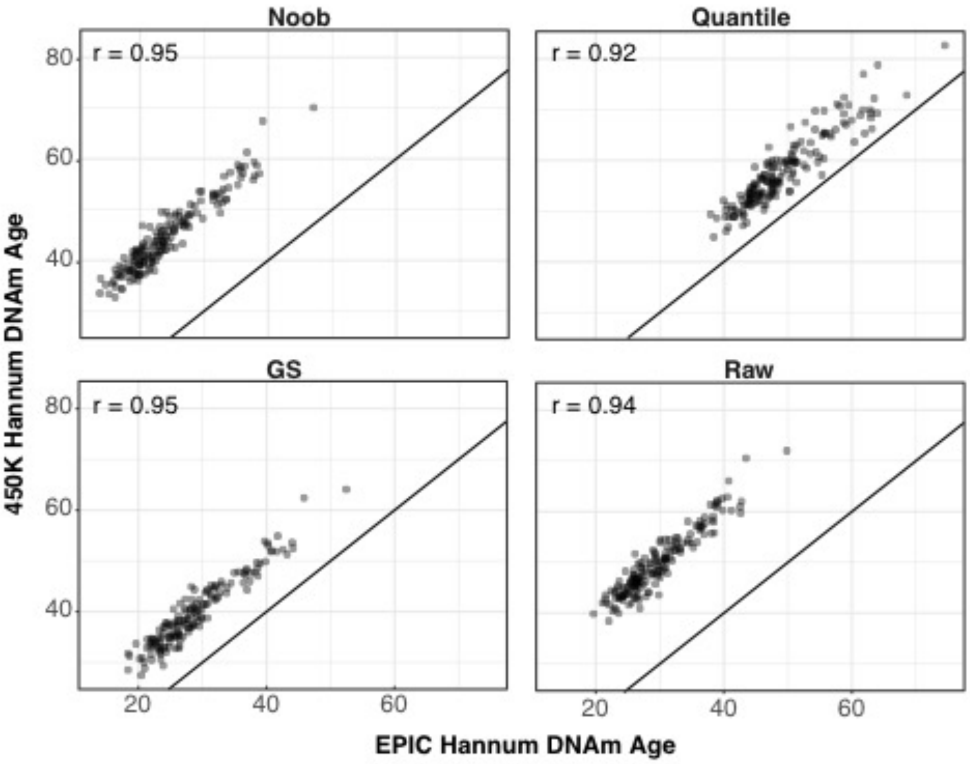


**Figure S4. Hannum DNA methylation age estimates for 450K (71 CpGs) versus EPIC (65 CpGs) for each preprocessed data type.**

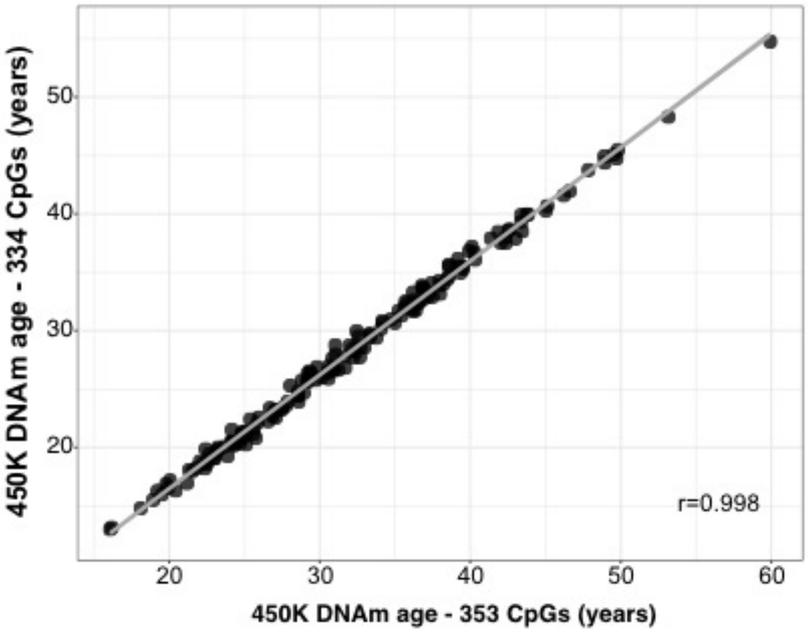


**Figure S5. DNA methylation (DNAm) age from a reduced epigenetic age predictor (334 CpGs) compared to the full epigenetic age predictor (353 CpGs) using the same 450K dataset.** DNAm age derived from a reduced raw 450K dataset with only probes present on the EPIC platform (334 clock sites) compared to DNAm age calculated from a full raw 450K dataset (353 CpGs).

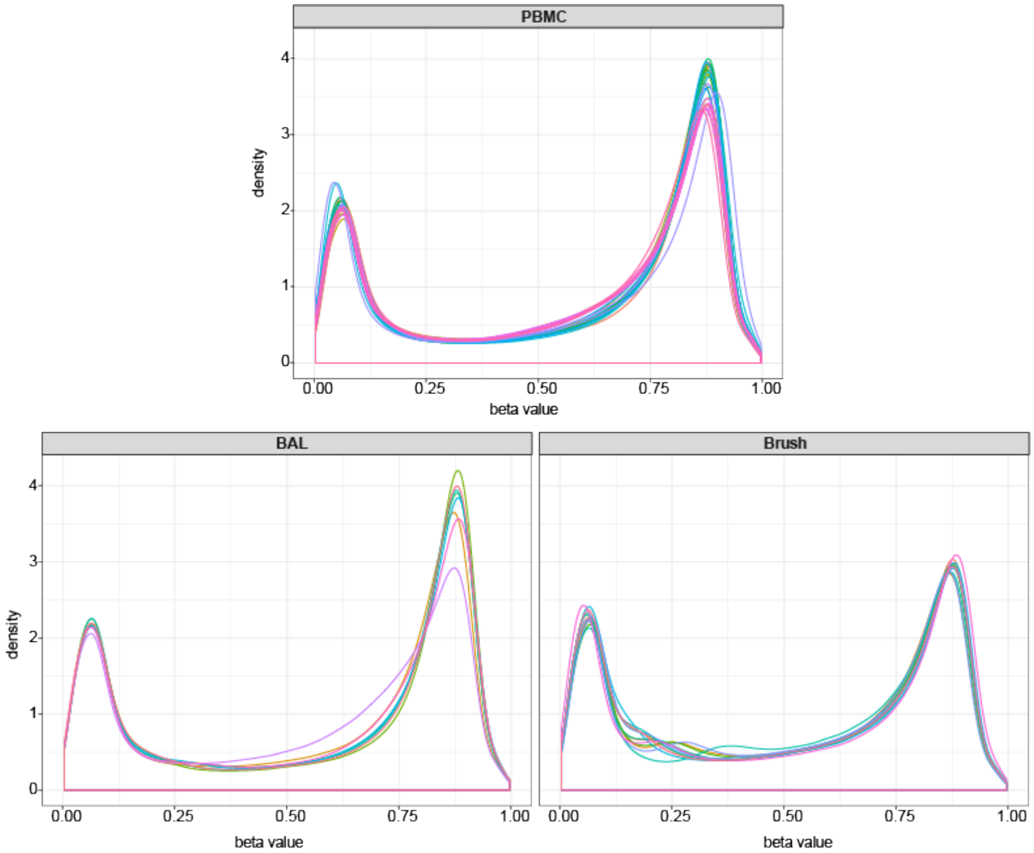


**Figure S6. Diesel Exhaust Study III EPIC DNA methylation beta-value distribution across 795,882 sites.** Each individual is represented by a color line. BAL = Bronchoalveolar lavage samples, PBMC = peripheral blood mononuclear cells, Brush = bronchial brushing samples.

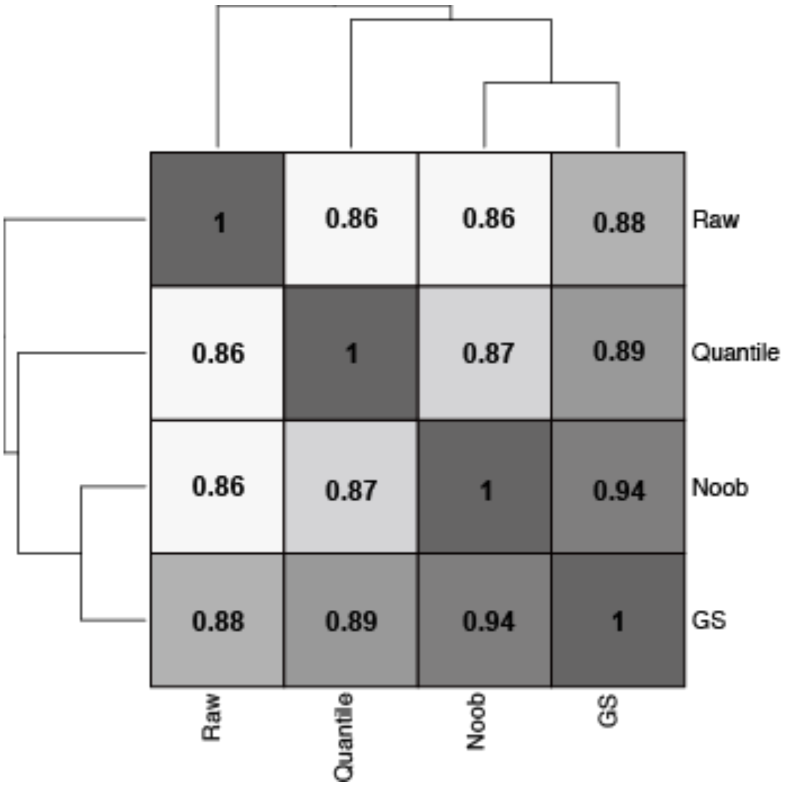


**Figure S7. Heat map of Kendall rank coefficients across preprocessing methods in EPIC data.**

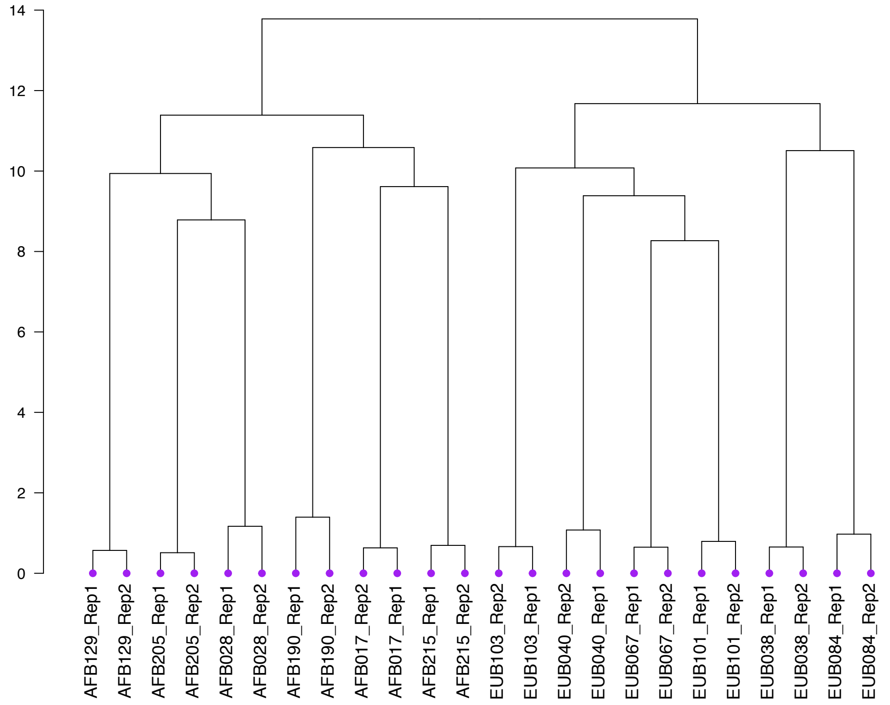


**Figure S8. Dendrogram of 59 single nucleotide polymorphic control probes of technical replicates from the EPIC array**.
